# (2*E*,6*E*)-2,6-Bis(2,6-dichloro­benzyl­idene)­cyclo­hexa­none

**DOI:** 10.1107/S1600536812006629

**Published:** 2012-02-17

**Authors:** Gholam Hossein Mahdavinia, Maryam Mirzazadeh, Vahid Amani, Behrouz Notash

**Affiliations:** a Department of Chemistry, Marvdasht Branch, Islamic Azad University, Marvdasht, Iran; b Department of Chemistry, Shahre-Rey Branch, Islamic Azad University, Tehran, Iran; c Department of Chemistry, Shahid Beheshti University, G.C., Evin, Tehran 1983963113, Iran

## Abstract

The title compound, C_20_H_14_Cl_4_O, was prepared by the reaction of 2,6-dichloro­benzaldehyde and cyclo­hexa­none. In the mol­ecule, the central cyclo­hexa­none ring adopts an envelope conformation, while the terminal benzene rings make a dihedral angle of 57.87 (9)°.

## Related literature

For background and applications of aryl­idene cyclo­alkanones, see: Deli *et al.* (1984 ▶); Nakano *et al.* (1987 ▶); Kawamata *et al.* (1996 ▶); Dimmock *et al.* (2003 ▶); Raj *et al.* (2003 ▶); Gangadhara (1995 ▶). For related structures, see: Yu *et al.* (2000 ▶); Zhou (2007 ▶).
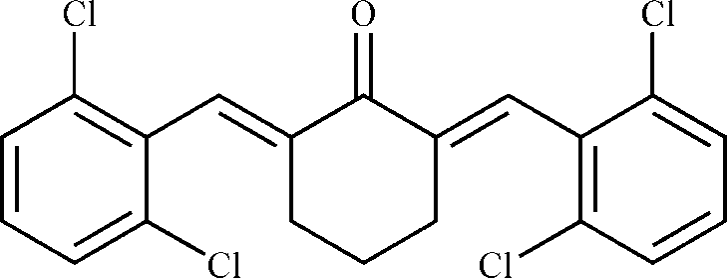


## Experimental

### 

#### Crystal data

C_20_H_14_Cl_4_O
*M* *_r_* = 412.11
Orthorhombic, 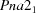

*a* = 17.917 (4) Å
*b* = 7.3094 (15) Å
*c* = 14.093 (3) Å
*V* = 1845.7 (7) Å^3^
*Z* = 4
Mo *K*α radiation
μ = 0.65 mm^−1^
*T* = 120 K
0.6 × 0.35 × 0.33 mm

#### Data collection

Stoe IPDS 2T diffractometer
13510 measured reflections
4946 independent reflections
4682 reflections with *I* > 2σ(*I*)
*R*_int_ = 0.043

#### Refinement

*R*[*F* ^2^ > 2σ(*F* ^2^)] = 0.031
*wR*(*F* ^2^) = 0.071
*S* = 1.04
4946 reflections
226 parameters
1 restraint
H-atom parameters constrained
Δρ_max_ = 0.25 e Å^−3^
Δρ_min_ = −0.20 e Å^−3^
Absolute structure: Flack (1983 ▶), 2369 Friedel pairs
Flack parameter: 0.01 (4)

### 

Data collection: *X-AREA* (Stoe & Cie, 2005 ▶); cell refinement: *X-AREA*; data reduction: *X-RED32* (Stoe & Cie, 2005 ▶); program(s) used to solve structure: *SHELXTL* (Sheldrick, 2008 ▶); program(s) used to refine structure: *SHELXTL*; molecular graphics: *SHELXTL*; software used to prepare material for publication: *SHELXTL*.

## Supplementary Material

Crystal structure: contains datablock(s) I, global. DOI: 10.1107/S1600536812006629/xu5466sup1.cif

Structure factors: contains datablock(s) I. DOI: 10.1107/S1600536812006629/xu5466Isup2.hkl

Supplementary material file. DOI: 10.1107/S1600536812006629/xu5466Isup3.cml

Additional supplementary materials:  crystallographic information; 3D view; checkCIF report

## Figures and Tables

**Fig. 1.**
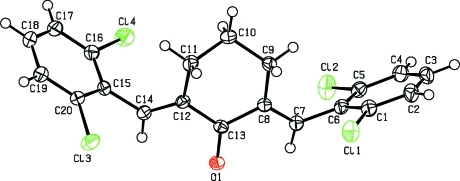
The molecular structure of the title molecule, with the atom-numbering scheme. Displacement ellipsoids are drawn at the 50% probability level.

## supplementary crystallographic information

### Comment

Cross-aldol condensation of aromatic aldehydes with cyclic ketones is an important protocol for the synthesis of arylidene cycloalkanones, which are very important precursors to potentially bioactive pyrimidine derivates (Deli *et al.*, 1984), intermediates for agrochemical, pharmaceuticals and perfumes (Nakano *et al.*, 1987), new organic material for nonlinear optical applications (Kawamata *et al.*, 1996), cytotoxic analogous (Dimmock *et al.*, 2003), bis-spiropyrrolidines (Raj *et al.*, 2003) and the units of liquid crystalline polymers (Gangadhara, 1995). Usually, this condensation process is catalyzed by strong acid or base.

In the molecule of the title compound, (Fig. 1), the bond lengths and angles are within normal ranges (Yu *et al.*, 2000; Zhou, 2007). A dihedral angle of 57.87 (9) A is found between the mean planes of the two benzene rings.

### Experimental

To a 10 ml solution of KOH (0.11 g) in ethanol at 313 K in a round bottom flask, cyclohexanone (5.0 mmol, 0.50 g) and 2,6-dichlorobenzaldehyde (10 mmol, 1.75 g) was added and the mixture was stirred for 2 min. The resulting product was then isolated by simple filtration from the reaction mixture and given washings with water to remove any trace of KOH remaining on the product. Yellow crystals, yield 97%, 1.98 g, m. p. 455–458 K.

### Refinement

All H atoms were positioned geometrically with C–H = 0.93–0.97 Å and constrained to ride on their parent atoms, with *U*_iso_(H) = 1.2*U*_eq_(C).

### Crystal data

**Table:**

C_20_H_14_Cl_4_O	*F*(000) = 840
*M**_r_* = 412.11	*D*_x_ = 1.483 Mg m^−^^3^
Orthorhombic, *P**n**a*2_1_	Mo *K*α radiation, λ = 0.71073 Å
Hall symbol: P 2c -2n	Cell parameters from 4949 reflections
*a* = 17.917 (4) Å	θ = 2.3–29.2°
*b* = 7.3094 (15) Å	µ = 0.65 mm^−^^1^
*c* = 14.093 (3) Å	*T* = 120 K
*V* = 1845.7 (7) Å^3^	Needle, yellow
*Z* = 4	0.6 × 0.35 × 0.33 mm

### Data collection

**Table:**

Stoe IPDS 2T diffractometer	4682 reflections with *I* > 2σ(*I*)
Radiation source: fine-focus sealed tube	*R*_int_ = 0.043
Graphite monochromator	θ_max_ = 29.2°, θ_min_ = 2.3°
rotation method scans	*h* = −24→24
13510 measured reflections	*k* = −8→10
4946 independent reflections	*l* = −19→19

### Refinement

**Table:**

Refinement on *F*^2^	Secondary atom site location: difference Fourier map
Least-squares matrix: full	Hydrogen site location: inferred from neighbouring sites
*R*[*F*^2^ > 2σ(*F*^2^)] = 0.031	H-atom parameters constrained
*wR*(*F*^2^) = 0.071	*w* = 1/[σ^2^(*F*_o_^2^) + (0.0318*P*)^2^ + 0.5994*P*] where *P* = (*F*_o_^2^ + 2*F*_c_^2^)/3
*S* = 1.04	(Δ/σ)_max_ = 0.001
4946 reflections	Δρ_max_ = 0.25 e Å^−^^3^
226 parameters	Δρ_min_ = −0.20 e Å^−^^3^
1 restraint	Absolute structure: Flack (1983), 2369 Friedel pairs
Primary atom site location: structure-invariant direct methods	Flack parameter: 0.01 (4)

### Special details

**Table:**

Geometry. All e.s.d.'s (except the e.s.d. in the dihedral angle between two l.s. planes) are estimated using the full covariance matrix. The cell e.s.d.'s are taken into account individually in the estimation of e.s.d.'s in distances, angles and torsion angles; correlations between e.s.d.'s in cell parameters are only used when they are defined by crystal symmetry. An approximate (isotropic) treatment of cell e.s.d.'s is used for estimating e.s.d.'s involving l.s. planes.
Refinement. Refinement of *F*^2^ against ALL reflections. The weighted *R*-factor *wR* and goodness of fit *S* are based on *F*^2^, conventional *R*-factors *R* are based on *F*, with *F* set to zero for negative *F*^2^. The threshold expression of *F*^2^ > σ(*F*^2^) is used only for calculating *R*-factors(gt) *etc*. and is not relevant to the choice of reflections for refinement. *R*-factors based on *F*^2^ are statistically about twice as large as those based on *F*, and *R*- factors based on ALL data will be even larger.

### Fractional atomic coordinates and isotropic or equivalent isotropic displacement parameters (Å^2^)

**Table:**

	*x*	*y*	*z*	*U*_iso_*/*U*_eq_	
Cl1	0.23181 (3)	1.40628 (7)	0.99333 (3)	0.03179 (11)	
Cl3	−0.14648 (3)	0.88978 (7)	0.82912 (3)	0.03028 (10)	
Cl4	0.05216 (3)	0.35099 (7)	0.89917 (4)	0.03460 (11)	
Cl2	0.30403 (3)	0.77001 (7)	1.16748 (4)	0.03902 (13)	
C12	0.04419 (9)	0.8310 (2)	0.91901 (11)	0.0196 (3)	
O1	0.04467 (7)	0.94480 (19)	1.07669 (9)	0.0237 (3)	
C6	0.27374 (10)	1.0891 (2)	1.07795 (12)	0.0211 (3)	
C13	0.07918 (9)	0.9234 (2)	1.00226 (12)	0.0185 (3)	
C7	0.19499 (9)	1.0268 (2)	1.07289 (12)	0.0199 (3)	
H7	0.1686	1.0178	1.1295	0.024*	
C9	0.19554 (9)	0.9844 (3)	0.89597 (12)	0.0253 (3)	
H9A	0.2490	0.9679	0.9032	0.030*	
H9B	0.1872	1.1026	0.8666	0.030*	
C15	−0.05043 (9)	0.6110 (2)	0.85940 (12)	0.0209 (3)	
C5	0.32884 (10)	0.9818 (3)	1.12039 (12)	0.0252 (4)	
C18	−0.11451 (11)	0.3989 (3)	0.71361 (15)	0.0317 (4)	
H18	−0.1354	0.3295	0.6651	0.038*	
C8	0.15938 (9)	0.9827 (2)	0.99274 (12)	0.0186 (3)	
C1	0.29745 (11)	1.2584 (3)	1.04280 (12)	0.0253 (4)	
C16	−0.02454 (10)	0.4355 (3)	0.83722 (14)	0.0246 (3)	
C10	0.16500 (10)	0.8350 (3)	0.83127 (13)	0.0278 (4)	
H10A	0.1873	0.8457	0.7688	0.033*	
H10B	0.1777	0.7157	0.8568	0.033*	
C19	−0.14239 (10)	0.5721 (3)	0.73292 (14)	0.0281 (4)	
H19	−0.1820	0.6191	0.6979	0.034*	
C11	0.08055 (10)	0.8530 (3)	0.82365 (12)	0.0260 (4)	
H11A	0.0681	0.9721	0.7978	0.031*	
H11B	0.0615	0.7605	0.7806	0.031*	
C3	0.42366 (10)	1.2027 (3)	1.08876 (13)	0.0318 (4)	
H3	0.4733	1.2393	1.0916	0.038*	
C14	−0.01489 (9)	0.7245 (2)	0.93404 (12)	0.0211 (3)	
H14	−0.0350	0.7210	0.9948	0.025*	
C20	−0.11038 (10)	0.6741 (3)	0.80531 (12)	0.0222 (3)	
C17	−0.05563 (11)	0.3289 (3)	0.76625 (15)	0.0295 (4)	
H17	−0.0373	0.2122	0.7541	0.035*	
C4	0.40302 (11)	1.0347 (3)	1.12538 (14)	0.0296 (4)	
H4	0.4384	0.9584	1.1530	0.036*	
C2	0.37123 (11)	1.3166 (3)	1.04810 (13)	0.0298 (4)	
H2	0.3850	1.4306	1.0246	0.036*	

### Atomic displacement parameters (Å^2^)

**Table:**

	*U*^11^	*U*^22^	*U*^33^	*U*^12^	*U*^13^	*U*^23^
Cl1	0.0347 (2)	0.0280 (2)	0.0326 (2)	−0.00606 (18)	−0.00506 (19)	0.0103 (2)
Cl3	0.0346 (2)	0.0290 (2)	0.0272 (2)	0.00852 (18)	−0.00735 (18)	−0.00764 (19)
Cl4	0.0329 (2)	0.0303 (2)	0.0406 (3)	0.00904 (18)	−0.0048 (2)	−0.0030 (2)
Cl2	0.0393 (2)	0.0295 (2)	0.0483 (3)	−0.0027 (2)	−0.0170 (2)	0.0103 (2)
C12	0.0205 (7)	0.0212 (8)	0.0172 (7)	0.0006 (6)	−0.0003 (6)	−0.0023 (6)
O1	0.0229 (6)	0.0316 (7)	0.0167 (5)	−0.0022 (5)	0.0025 (4)	−0.0034 (5)
C6	0.0233 (7)	0.0252 (8)	0.0147 (6)	−0.0025 (6)	−0.0014 (6)	−0.0006 (6)
C13	0.0185 (7)	0.0214 (7)	0.0157 (7)	0.0020 (6)	0.0008 (6)	0.0017 (6)
C7	0.0214 (7)	0.0217 (7)	0.0166 (7)	−0.0015 (6)	−0.0011 (6)	0.0020 (6)
C9	0.0218 (8)	0.0344 (10)	0.0197 (7)	−0.0056 (7)	0.0038 (7)	−0.0032 (8)
C15	0.0188 (7)	0.0219 (8)	0.0219 (7)	−0.0041 (6)	0.0023 (6)	−0.0011 (6)
C5	0.0273 (9)	0.0279 (9)	0.0204 (8)	−0.0009 (7)	−0.0027 (7)	−0.0023 (7)
C18	0.0259 (9)	0.0345 (10)	0.0348 (10)	−0.0081 (8)	0.0025 (7)	−0.0151 (9)
C8	0.0196 (7)	0.0193 (7)	0.0169 (6)	−0.0001 (6)	0.0001 (6)	0.0010 (6)
C1	0.0267 (8)	0.0313 (9)	0.0179 (7)	−0.0039 (7)	−0.0008 (6)	0.0017 (7)
C16	0.0228 (7)	0.0228 (8)	0.0283 (9)	−0.0013 (6)	0.0026 (7)	−0.0026 (7)
C10	0.0276 (8)	0.0360 (10)	0.0197 (7)	−0.0038 (7)	0.0050 (7)	−0.0068 (8)
C19	0.0233 (8)	0.0340 (10)	0.0270 (9)	−0.0018 (7)	−0.0015 (7)	−0.0090 (8)
C11	0.0289 (8)	0.0331 (10)	0.0160 (7)	−0.0077 (7)	0.0011 (7)	−0.0018 (7)
C3	0.0220 (8)	0.0518 (12)	0.0216 (8)	−0.0093 (8)	0.0008 (7)	−0.0069 (8)
C14	0.0212 (7)	0.0233 (8)	0.0187 (7)	−0.0006 (7)	−0.0001 (6)	−0.0014 (6)
C20	0.0217 (8)	0.0232 (8)	0.0216 (8)	−0.0014 (7)	0.0017 (6)	−0.0040 (6)
C17	0.0281 (9)	0.0241 (9)	0.0362 (10)	−0.0048 (7)	0.0066 (8)	−0.0085 (8)
C4	0.0232 (8)	0.0412 (11)	0.0243 (8)	0.0021 (8)	−0.0063 (7)	−0.0062 (8)
C2	0.0299 (9)	0.0386 (10)	0.0210 (8)	−0.0137 (8)	0.0032 (7)	0.0017 (8)

### Geometric parameters (Å, º)

**Table:**

Cl1—C1	1.743 (2)	C5—C4	1.386 (3)
Cl3—C20	1.7370 (19)	C18—C17	1.388 (3)
Cl4—C16	1.7414 (19)	C18—C19	1.388 (3)
Cl2—C5	1.742 (2)	C18—H18	0.9300
C12—C14	1.331 (2)	C1—C2	1.391 (3)
C12—C13	1.492 (2)	C16—C17	1.385 (3)
C12—C11	1.502 (2)	C10—C11	1.523 (3)
O1—C13	1.228 (2)	C10—H10A	0.9700
C6—C5	1.396 (3)	C10—H10B	0.9700
C6—C1	1.399 (3)	C19—C20	1.388 (2)
C6—C7	1.484 (2)	C19—H19	0.9300
C13—C8	1.507 (2)	C11—H11A	0.9700
C7—C8	1.337 (2)	C11—H11B	0.9700
C7—H7	0.9300	C3—C2	1.380 (3)
C9—C8	1.510 (2)	C3—C4	1.382 (3)
C9—C10	1.524 (3)	C3—H3	0.9300
C9—H9A	0.9700	C14—H14	0.9300
C9—H9B	0.9700	C17—H17	0.9300
C15—C20	1.396 (2)	C4—H4	0.9300
C15—C16	1.399 (3)	C2—H2	0.9300
C15—C14	1.483 (2)		
			
C14—C12—C13	118.29 (15)	C15—C16—Cl4	118.34 (14)
C14—C12—C11	123.35 (15)	C11—C10—C9	109.69 (15)
C13—C12—C11	118.23 (14)	C11—C10—H10A	109.7
C5—C6—C1	115.73 (17)	C9—C10—H10A	109.7
C5—C6—C7	121.37 (17)	C11—C10—H10B	109.7
C1—C6—C7	122.89 (16)	C9—C10—H10B	109.7
O1—C13—C12	121.19 (15)	H10A—C10—H10B	108.2
O1—C13—C8	121.32 (16)	C18—C19—C20	119.04 (18)
C12—C13—C8	117.45 (14)	C18—C19—H19	120.5
C8—C7—C6	124.63 (15)	C20—C19—H19	120.5
C8—C7—H7	117.7	C12—C11—C10	111.02 (15)
C6—C7—H7	117.7	C12—C11—H11A	109.4
C8—C9—C10	112.35 (15)	C10—C11—H11A	109.4
C8—C9—H9A	109.1	C12—C11—H11B	109.4
C10—C9—H9A	109.1	C10—C11—H11B	109.4
C8—C9—H9B	109.1	H11A—C11—H11B	108.0
C10—C9—H9B	109.1	C2—C3—C4	120.58 (18)
H9A—C9—H9B	107.9	C2—C3—H3	119.7
C20—C15—C16	115.85 (16)	C4—C3—H3	119.7
C20—C15—C14	122.20 (16)	C12—C14—C15	123.77 (15)
C16—C15—C14	121.93 (16)	C12—C14—H14	118.1
C4—C5—C6	122.88 (19)	C15—C14—H14	118.1
C4—C5—Cl2	118.28 (15)	C19—C20—C15	122.83 (18)
C6—C5—Cl2	118.83 (14)	C19—C20—Cl3	118.41 (14)
C17—C18—C19	120.36 (18)	C15—C20—Cl3	118.76 (13)
C17—C18—H18	119.8	C16—C17—C18	118.97 (18)
C19—C18—H18	119.8	C16—C17—H17	120.5
C7—C8—C13	116.69 (15)	C18—C17—H17	120.5
C7—C8—C9	123.82 (15)	C3—C4—C5	119.07 (19)
C13—C8—C9	119.47 (14)	C3—C4—H4	120.5
C2—C1—C6	122.69 (18)	C5—C4—H4	120.5
C2—C1—Cl1	118.25 (16)	C3—C2—C1	119.02 (19)
C6—C1—Cl1	119.03 (14)	C3—C2—H2	120.5
C17—C16—C15	122.94 (18)	C1—C2—H2	120.5
C17—C16—Cl4	118.69 (15)		
			
C14—C12—C13—O1	−20.1 (3)	C14—C15—C16—Cl4	−0.5 (2)
C11—C12—C13—O1	163.96 (17)	C8—C9—C10—C11	55.7 (2)
C14—C12—C13—C8	157.57 (16)	C17—C18—C19—C20	0.2 (3)
C11—C12—C13—C8	−18.4 (2)	C14—C12—C11—C10	−132.96 (18)
C5—C6—C7—C8	−112.5 (2)	C13—C12—C11—C10	42.8 (2)
C1—C6—C7—C8	68.8 (3)	C9—C10—C11—C12	−61.2 (2)
C1—C6—C5—C4	−2.0 (3)	C13—C12—C14—C15	−173.89 (16)
C7—C6—C5—C4	179.24 (17)	C11—C12—C14—C15	1.8 (3)
C1—C6—C5—Cl2	179.18 (13)	C20—C15—C14—C12	−92.9 (2)
C7—C6—C5—Cl2	0.4 (2)	C16—C15—C14—C12	85.4 (2)
C6—C7—C8—C13	−179.65 (16)	C18—C19—C20—C15	0.7 (3)
C6—C7—C8—C9	2.1 (3)	C18—C19—C20—Cl3	−179.49 (15)
O1—C13—C8—C7	12.3 (2)	C16—C15—C20—C19	−0.8 (3)
C12—C13—C8—C7	−165.39 (16)	C14—C15—C20—C19	177.55 (17)
O1—C13—C8—C9	−169.41 (17)	C16—C15—C20—Cl3	179.37 (13)
C12—C13—C8—C9	12.9 (2)	C14—C15—C20—Cl3	−2.3 (2)
C10—C9—C8—C7	146.11 (18)	C15—C16—C17—C18	0.8 (3)
C10—C9—C8—C13	−32.1 (2)	Cl4—C16—C17—C18	−176.99 (15)
C5—C6—C1—C2	0.9 (3)	C19—C18—C17—C16	−0.9 (3)
C7—C6—C1—C2	179.69 (17)	C2—C3—C4—C5	0.2 (3)
C5—C6—C1—Cl1	−177.17 (13)	C6—C5—C4—C3	1.4 (3)
C7—C6—C1—Cl1	1.6 (2)	Cl2—C5—C4—C3	−179.70 (14)
C20—C15—C16—C17	0.1 (3)	C4—C3—C2—C1	−1.2 (3)
C14—C15—C16—C17	−178.32 (17)	C6—C1—C2—C3	0.6 (3)
C20—C15—C16—Cl4	177.86 (13)	Cl1—C1—C2—C3	178.73 (14)
